# Addressing Ethical, Legal, and Social Issues on Artificial Intelligence Together With Patients' Perspectives: Prerequisites for Responsible Social Implementation in Endoscopy

**DOI:** 10.1111/den.70168

**Published:** 2026-05-14

**Authors:** Yosuke Tsuji, Mitsuhiro Fujishiro

**Affiliations:** ^1^ Department of Gastroenterology Graduate School of Medicine, The University of Tokyo Tokyo Japan

Artificial intelligence (AI) has advanced rapidly in recent years and is increasingly integrated into many aspects of daily life and modern medical practice. Gastrointestinal (GI) endoscopy is no exception. Numerous studies have demonstrated the usefulness of AI‐assisted colonoscopy, particularly computer‐aided detection (CADe), which has been consistently associated with improved adenoma detection rate (ADR) [1]. These data indicate that CADe is becoming one of the most promising applications of AI in the clinical endoscopy field. Further, several investigations have suggested that AI‐supported colonoscopy can be cost‐effective by contributing to a reduction in long‐term colorectal cancer‐related mortality [2].

In contrast, evidence supporting computer‐aided diagnosis (CADx) remains limited. Although CADx systems offer the potential to reduce unnecessary polypectomies through real‐time optical diagnosis [3], current data have not yet reached the level required for confident clinical application. Reflecting this, the position statement issued by the Japanese Gastroenterological Endoscopy Society (JGES) notes that although CADe may increase ADR, the evidence supporting CADx remains insufficient, even though its clinical potential is undeniable [4]. This gap between technological promise and confirmatory evidence underscores the current stage of AI implementation in endoscopy.

The situation in upper GI endoscopy is not the same; evidence has not been so sufficient as in the colonoscopy field. Some reports suggest improved detection of gastric or esophageal neoplasia with AI support, but the evidence base remains far smaller than in colonoscopy. As a result, the JGES position statements conclude that neither CADe nor CADx currently has sufficient evidence to support definitive recommendations in upper GI settings [4]. In addition, recent WEO position statements for gastric cancer conclude that AI is still controversial in terms of cost‐effectiveness despite possible increasing diagnostic yields [5]. Larger and more rigorous studies will be needed to clarify the true utility of AI in upper GI endoscopy.

As new technologies emerge, both developers and early adopters tend to accelerate their introduction into clinical practice. However, responsible dissemination requires careful consideration of ethical, legal, and social issues (ELSI). ELSI was originally formulated during the Human Genome Project to provide a structured framework for understanding the effects of genome research on society and establishing governance [6]. Its relevance has since expanded far beyond genetics, and AI‐assisted endoscopy represents a field in which ELSI considerations are essential.

Ethical questions include the anonymization and proper management of endoscopic image data used to train AI systems, as well as clarification of data ownership. Legal issues arise when AI errors influence clinical decisions and cause patient harm. Determining responsibility—whether it lies with the physician, the hospital, or the manufacturer—remains an unresolved challenge. Social issues add further complexity. Implementing AI requires substantial financial investment, raising the possibility that access may initially be limited to resource‐rich institutions, potentially widening healthcare disparities.

Addressing these issues requires a commitment to patient and public involvement (PPI). PPI emphasizes that not only healthcare providers but patients or the public should participate in various stages of novel research (research conducted with or by patients, rather than to, about, or for them) [7]. A recent international survey conducted by Ahmad et al. under the World Endoscopy Organization (WEO) AI Ad Hoc Committee exemplifies this approach [8]. Their findings show broad support for AI implementation among both clinicians and patients. Notably, both groups agree that ultimate diagnostic responsibility should remain with physicians, suggesting that human judgment remains central even as AI tools become more advanced.

The survey also revealed differences in perceptions of liability. Clinicians tended to attribute responsibility for AI‐related errors to individual physicians or endoscopists, whereas many patients regarded the hospital as primarily responsible. This discrepancy demonstrates the necessity of establishing coherent medicolegal frameworks before the widespread adoption of AI in clinical endoscopy. Ahmad et al. have recently developed 10 consensus statements through a Delphi process addressing ethical and legal issues in AI‐assisted endoscopy [9]. Their work represents a genuine embodiment of the ELSI framework and highlights the importance of structured guidance and transparent policymaking as AI evolves.

Another consideration is the demographic limitation of the survey population. Many of the patient participants were in their 20s–40s, whereas most patients undergoing endoscopy in routine clinical settings are notably older. Therefore, we should bear in mind that the younger generation's opinions were reflected in this survey, especially for patients. When contemplating social implementation of AI, it is essential to recognize the perspectives of older patients.

Technological progress in AI continues at a remarkable pace. Despite ongoing challenges such as false positives and false negatives, rapid advancements—mirroring those seen in generative AI—suggest that future AI systems may achieve far higher levels of accuracy. Such improvement could fundamentally shift the clinical landscape. If AI eventually surpasses expert human performance, new questions will emerge. Will physicians or hospitals remain primarily responsible for diagnostic errors? Could failure to use AI itself be regarded as a deviation from standard care? And will concerns about clinician de‐skilling that accompany the use of AI become more pronounced [10], or will they diminish in relevance as AI becomes increasingly integrated into clinical practice?

These questions do not yet have answers. However, one point remains clear: at this moment, decisions regarding how AI should be used are ultimately made by humans. The future direction of AI‐assisted endoscopy depends not only on technological capabilities but also on collective decisions balancing innovation, safety, fairness, and patient‐centeredness. As we stand at the threshold of a new era in endoscopic medicine, it is essential to approach AI with both optimism and responsibility. We hope the article by Ahmad et al. and this editorial will be the starting point for future researchers and administrators.

AI holds great potential to transform GI endoscopy. Its successful integration will require thoughtful examination of ethical, legal, and social considerations, robust scientific evidence, and a shared commitment to maintaining patient trust. The core values of medicine will not change—we must remember that.

## Author Contributions

Y.T. drafted the initial manuscript, and both Y.T. and M.F. revised it critically for important intellectual content and approved the final version of the manuscript.

## Funding

The authors have nothing to report.

## Conflicts of Interest

Y.T. and M.F. have received a speaker honorarium from AI Medical Service Inc.

## Linked Article

This article is linked to Ahmad et al. papers. To view this article, visit https://doi.org/10.1111/den.70123.
